# Safety and efficacy of “on-demand” tramadol in patients with premature ejaculation: an updated meta-analysis

**DOI:** 10.1590/S1677-5538.IBJU.2020.0561

**Published:** 2020-09-10

**Authors:** Aditya Prakash Sharma, Gopal Sharma, Shantanu Tyagi, Sudheer K. Devana, Ravimohan S. Mavuduru, Girdhar S. Bora, Shrawan K. Singh

**Affiliations:** 1 PGIMER Department of Urology Chandigarh India Department of Urology, PGIMER, Chandigarh, India

**Keywords:** Tramadol, Premature Ejaculation, Systematic Reviews as Topic

## Abstract

**Introduction:**

Tramadol has been used for the treatment of premature ejaculation, however, the studies published for the same are not well designed. The primary objective of this study was to explore the literature pertaining to the use of tramadol in patients with PE to determine its safety and efficacy in this population.

**Materials ande methods:**

Systematic literature search of various electronic databases was conducted to include all the randomized studies and quasi-randomized studies. Standard PRISMA (Preferred reporting Items for Systematic reviews and Meta-analysis) guidelines were pursued for this review and study protocol was registered with PROSPERO (CRD42019123381).

**Results:**

Out of 9 studies included in this review, 5 were randomized controlled trials, and rests of the 4 studies were quasi-randomized studies. Tramadol resulted in significantly higher improvement of IELT with the mean difference (MD) of 139.6 seconds and confidence interval (CI) 106.5-172.6 seconds with a p-value of p <0.00001. All dosages except 25mg fared well as compared to placebo. Tramadol fared better than placebo at 1 month, 2 months, and 3 months after initiation of therapy as compared to the placebo. Tramadol group had reported a significantly higher number of adverse events with treatment as compared to placebo but none of them were serious.

**Conclusion:**

Tramadol appears to be an effective drug for the management of PE with a low propensity for serious adverse events. However, evidence obtained from this study is of low to moderate quality. Furthermore, effective dose and duration of therapy remain elusive.

## INTRODUCTION

Premature ejaculation (PE) is one of the most commonly encountered sexual dysfunction in males. It has been defined by the International society of sexual medicine (ISSM) guidelines as “male sexual dysfunction characterized by ejaculation within about one minute of vaginal penetration (lifelong PE) or a reduction in latency time to <3 minutes (secondary PE) and having negative personal consequences” (1). The treatment of PE varies including behavioral and pharmacological therapies. Various off-label pharmacological treatments for PE include use of local anesthetic sprays, selective serotonin uptake inhibitors (SSRI's) such as paroxetine (2), dapoxetine (3), citalopram, sertraline, trazodone, fluvoxamine and fluoxetine, tricyclic antidepressants such as clomipramine (4), opioid analgesics such as tramadol. (5, 6). SSRIs and clomipramine have been studied in both daily and on-demand settings and has been proven to be more effective than the placebo or control group (5). On-demand use of a particular pharmacological agent is more convenient for the patients; it also reduces tachyphylaxis and adverse effects associated with daily use (5).

On-demand tramadol has been used for the treatment of PE. The mechanism of action of tramadol is not clear but had been hypothesized that tramadol activates opioid receptors and inhibits the uptake of serotonin and nor-adrenaline. Tramadol has been used in various dosages and for a variable duration. The use has been found to be efficacious in various studies however the side effect profile and addiction potential of the drug has limited its use. The main aim of this study was to systematically review the existing literature and perform a meta-analysis evaluating the effectiveness of tramadol in on-demand setting as compared to placebo or other treatments.

## MATERIALS AND METHODS

### 

#### Study design

With this systematic review and metanalysis, we intended to summarize the current literature on the safety and efficacy of tramadol in patients with lifelong PE. Prior to initiation of the study, the protocol was registered under PROSPERO on Nov 2018 (CRD42019123381). Present review was conducted in conformity with current Preferred Reporting Items for Systematic reviews and Meta-analysis (PRISMA) Guidelines (7).

#### Search strategy

Two study authors (AS & GS) independently performed the database search to identify articles pertaining to the use of on-demand tramadol in PE. We used Pubmed, Scopus, Embase, and Web of Science databases to carry out the literature search from their date of inception till January 2020. The following search filters were applied Language [English] and [Human]. Third author (ST) help was sought to reach agreement regarding inclusion or exclusion of any article during different stages of the review in case of discrepancy.

We used the PICO (Patient/Population, Intervention, Control, Outcome) framework to design the strategy for evidence synthesis.

- Patient/population - Premature ejaculation
- Intervention - Tramadol
- Control - Placebo or control
- Outcome - Intravaginal ejaculation latency time (IELT)

For the literature search, we used the following keywords Tramadol and premature ejaculation OR PE. The last literature search was conducted on 28^th^ January 2020. All the search results were then transferred on to a review manager and all the duplicates were identified and removed.

#### Selection criteria

Initially, two study authors (GS & AS) assessed the titles, followed by abstracts of the relevant articles obtained from the online database search. Articles containing data on the use of tramadol in premature ejaculation were selected for full-text review. Based on inclusion and exclusion criteria, studies were selected for eligibility for full article review. Studies conducted in randomized or quasi-randomized fashion describing the use of “on-demand” tramadol in a comparative setting with placebo or other drugs were included. Studies conducted on the daily use of tramadol, lacking a comparative group such as placebo or controls were excluded. Studies not providing primary outcomes on the basis of IELT were also excluded. Any disagreement regarding inclusion or exclusion of a study was resolved by arbitration among the three study authors [GS, ST, and AS].

#### Outcomes

The primary outcome measure used for this study was IELT after treatment duration. Treatment duration varied across the studies, thus we performed combined analysis and analysis according to the duration of therapy. A Comparison of tramadol to other forms of therapy was also done wherever data was available. Apart from IELT, different studies have used different outcome measures such as PEP (premature ejaculation profile), IIEF score (International index of erectile function), AIPE (Arabic index of premature ejaculation), sexual satisfaction and control over ejaculation. Since the data on these secondary outcomes measures is heterogeneously reported across the included studies these were included only for narrative data synthesis. For the safety profile of tramadol, comparison of tramadol to control group was performed by extracting data from studies where treatment-related adverse events were reported.

#### Data extraction

From the included studies, data were extracted by two review authors (GS & AS) in a predefined format for the final analysis. Quantitative data synthesis was performed for all the continuous variables obtained and expressed as mean and standard deviation. Predefined data extraction template included first author name, year of publication, country of origin, type of study, the definition of PE, drugs used during the study, treatment duration, study protocol, and outcome measures. Following the completion of data extraction, data were compared for consistency and any discrepancy was resolved by reassessing the data and arbitration by the third author (ST).

#### Quality assessment

Cochrane risk of bias assessment tool was used for quality assessment that scrutinizes a study across seven domains (8). Finally, studies are graded as “high risk”, “low risk” or ‘unclear” risk of bias’ across the seven domains. Two study authors conducted the quality or risk of bias assessment independently and any discrepancy was settled after arbitration with a third author (ST).

### Satistical Analysis

For continuous variables, mean and standard deviation was extracted from the included studies. In case data were expressed as median or range, mean and standard deviation was estimated using method described by the Hozo et al. (9) and used in our previous studies (10, 11). Pooled mean differences (MD) with their 95% confidence interval (95% CI) were estimated. For dichotomous variables, statistical heterogeneity was tested using chi^2^ and I^2^ tests. A p value <0.10 was used to indicate heterogeneity and in the absence of statistical heterogeneity the fixed-effects model (Mantel-Haenszel method) was used. In the presence of a statistically significant heterogeneity, random effects model was used. Statistical analysis was accomplished using the RevMan 5.2^TM^ software (the Cochrane collaboration, Copenhagen, Denmark) and p-value <0.05 was used to define statistical significance.

## RESULTS

Search strategy and selection

**Figure-1:**
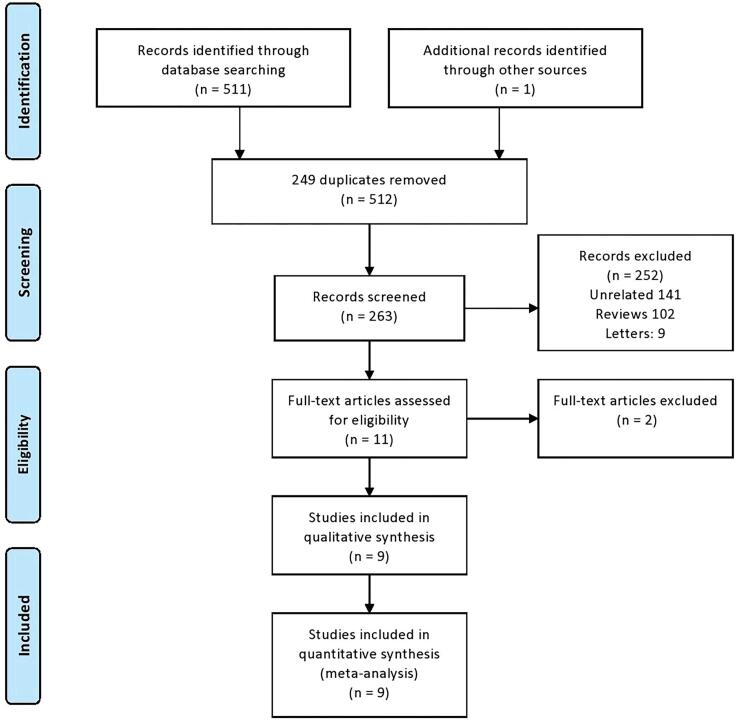
PRISMA flow-chart depicting search strategy used during this review.

An extensive literature search was done using four databases Pubmed, Scopus, Embase, and Web of science. A total of 512 citations were retrieved into a citation manager. 249 duplicate citations were removed and 263 articles were screened for eligibility. Out of these 263 articles, 252 articles were excluded for various reasons (Figure-1). Eleven articles were selected for full-text review. Two articles were excluded following full-text review as one did not contain desired groups of comparison and the other full-text was in Chinese (12). A total of 9 articles were included in this study (12–21).

### 

#### Study characteristics

Out of 9 studies included in this review, 5 were RCT, and the rest of the 4 studies were quasi-randomized studies. Duration of therapy varied from 4-20 weeks across the studies. Various doses of tramadol were tested including 25mg, 50mg, 62mg, 89mg, and 100mg. IELT has been the primary outcome measure used across all the studies whereas various secondary outcome measures were used in different studies. Most of the studies have used non-validated secondary outcome measures with variable scoring systems therefore, the quantitative synthesis of such data was not possible and they have been provided in Table-1.

**Table-1:** Characteristics of the studies included in the review.

Author	Year/Country	Type of study	Definition of PE	Study protocol	Outcome measures	Secondary outcomes measures results
Safarinejad et al. (13) Tramadol 50mg (29) Placebo (28) 8 weeks	2006 Iran	RCT	IELT <2mins for >90% of the coitus	-Age group 20-52 -Randomly assigned in two groups. -Drug taken 2 hours before sexual activity. -Patient followed 2 weekly till 8 weeks.	IELT Sexual satisfaction domain values of IIEF	Mean intercourse satisfaction domain values of the IIEF at 8 weeks in tramadol and plaeco group were 14 and 10 respectively.
Salem et al. (14) Tramadol 25 mg (60) Placebo (60) 8 weeks	2008 USA	Prospective single blind Placebo controlled Cross over study	DSM IV TR (IELT <2 mins in >80 % of coitus acts)	-4 weeks of test period followed by 8 weeks of treatment duration -I week drug wash out period followed by crossover of therapy for 8 weeks again -Drug taken 1-2 hours before act.	IELT Satisfaction over control of ejaculation Satisfaction with sexual function	59/60 patients reported satisfactory control over ejaculation and significant benefits in sexual satisfaction
Bar-or et al. (15) Tramadol 62mg ODT (206) Tramadol 89mg ODT (198) Placebo (200) 12 weeks	2012 Multicentre	RCT	DSM IV TR IELT <2 mins	Baseline 3 week screening period Followed by 3 week single blind placebo lead-in period Followed by 12 weeks of double blind treatment Drugs taken 2-8 hours before sex.	IELT PEP	NA
Kaynar et al. (16) Tramadol 25 mg (30) Placebo (30) 8 weeks	2012 Turkey	Single blind placebo controlled Crossover study	IELT <1min for >90% of the coitus	Drug taken 2 hours before sex. Two groups containing 30 patients were given either placebo or tramadol first followed by cross over	IELT Ability to control ejacuation Sexual satisfaction scores	Ejaculation control ability Tramadol group 2.83 vs. 1.5 for placebo Sexual satisfaction score Tramadol group 2.77 vs. 1.33 for placebo
Eassa et al. (17) Tramadol 25mg, 50mg and 100 mg Placebo lead in period	2012 Egypt	RCT	NA	Initially all patients were given placebo for 4 weeks followed by randomization into 3 weeks to receive different doses of tamadol for 24 weeks. Drug was given 2–3 h before sex	IELT Satisfaction and control of ejaculation	90% (270/300)) treated with tramadol reported an increase in penile rigidity. Tramadol 100 mg group had higher side effects. However none of the side effects were serious
Khan et al. (18) Tramadol 100 mg 4 weeks daily and 4 weeks on demand	2013 India	RCT	DSM IV TR IELT <2 mins	One group of patients received 100 mg daily of tramadol for 4 weeks and then on demand every 2 or 8 h before coitus for next 4 weeks. Second group patients received placebo for 4 weeks daily and on demand for next 4 weeks 2 or 8 h prior to coitus.	IELT	NA
Gameel et al. (19) Tramadol 50 mg (29) Sildenafil 50 mg (30) Paroxetine (28) Local anesthetic (30) Placebo (27) 4 weeks	2013 Egypt	Single blind placebo controlled	IELT <2 min in >75% of sexual acts over a 2 week period	4 week run in period followed by 4 weeks of treatment period. Tramadol given 2 hours before sex whereas sildenafil, paroxetine and local anesthetic given 1 hour, 4 hours and 15 mins prior to the act	IELT Sexual satisfaction score	Sexual satisfaction score was 3.7 in tramadol group vs. 1.18 in placebo group.
Kurkar et al. (20) Tramadol 50mg and 100mg Placebo For 8 weeks	2015 Egypt	RCT with crossover design	NA	Study subjects were randomized into 3 groups to receive three treatments. Group 1: 50 mg tramadol, placebo and tramadol 100mg Group 2: 100 mg tramadol, placebo and 50mg tramadol Group 3: 100mg tramadol, 50 mg tramadol and placebo.	IELT	NA
Hamidi-Madani et al. (21) Tramadol 50mg Paroxetine 20mg Placebo 12 weeks	2018 Iran	RCT	IELT<1 min	Study subjects randomized into three groups after a 3 weeks lead-in period and subjects were reassessed after 12 weeks. Drug taken 2-3 hours prior to act.	IELT PEP	PEP score at 12 weeks were 13.3, 11.3 and 9.97 for tramadol, paroxetine and placebo respectively.

#### Definition of PE

The definition of PE has been varied across the studies, some studies have described it as IELT less than 2 mins whereas others have taken less than 1 min. It is defined by ISSM guidelines as “male sexual dysfunction characterized by ejaculation within about one minute of vaginal penetration (lifelong PE) or a reduction in latency time to <3 minutes (secondary PE) and having negative personal consequences”. Diagnostic and Statistical Manual of Mental Disorders-IV (DSM IV TR) (22) text revision defined it as ejaculation occurring within 2 mins and ISSM (23) defined it at less than one min. Latest DSM V (24) and ISSM 2014 guidelines (1) both defined PE as less than 1 min. DSM V has also described PE should have been present for at least 6 months and on 75-100% of the times (24).

#### Quality assessment

**Figure-2:**
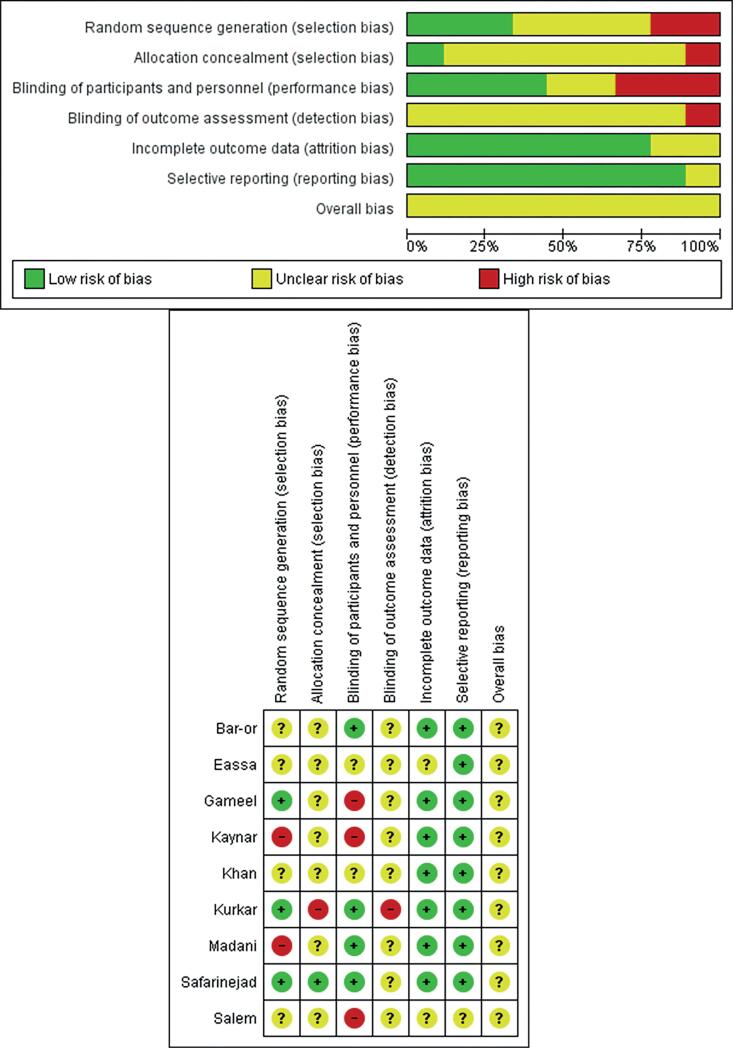
Risk of bias assessment summary and graph.

The overall quality of studies in this review appears to be of low to medium quality. Randomization technique was described only in three studies and allocation concealment was done only in one study. Blinding of both the participants and the investigator was done only in 4 studies, 3 studies were single-blind and 2 studies didn't mention about double-blinding. Overall summary of the risk of bias is provided in Figure-2. Publication bias was assessed using Egger's test for which the p-value was 0.3 i.e. there is no publication bias.

#### IELT

**Figure-3:**
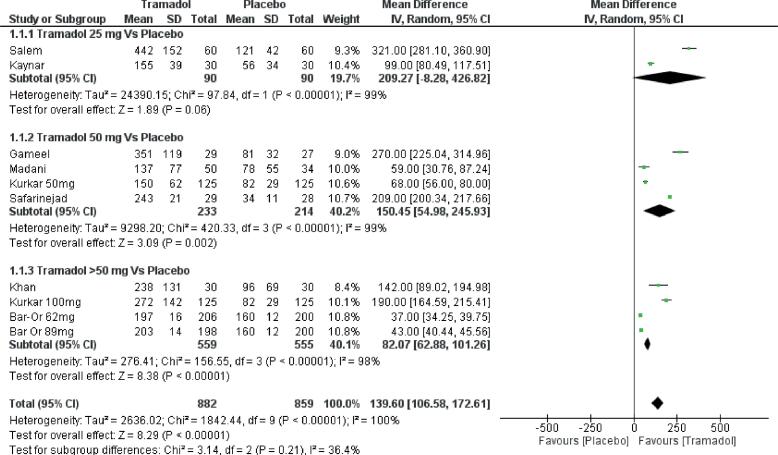
Forest plot depicting comparison of various dosages of Tramadol to placebo in Premature ejaculation.

Overall comparison of tramadol was done against placebo or no treatment irrespective of the dose and duration of the therapy. Tramadol resulted in significantly higher improvement of IELT with the mean difference (MD) of 139.6 seconds and confidence interval (CI) 106.5-172.6 seconds with a p-value of p <0.00001 (Figure-3). There was high heterogeneity across the studies included in the final analysis thus random effect model was used for meta-analysis. Various subgroup analyses according to dose and duration of treatment were also done in this study.

#### Dose variation

**Tramadol 25mg, 50mg and >50mg vs. placebo**

Kaynar et al. (25) and Salem et al. (26) had compared tramadol 25mg to placebo and the two groups were not significantly different (MD=209.2, CI-8.2 to 426.8, p=0.06). Upon comparing studies reporting data at 50mg [MD 150.4 CI (54.9, 245.9), p=0.002] and >50mg (62mg, 89mg and 100mg) [MD 82.07 CI (62.8, 101.2), p <0.00001] tramadol to placebo the dosages fared better as compared to placebo (Figure-3).

#### Duration of treatment

**Figure-4:**
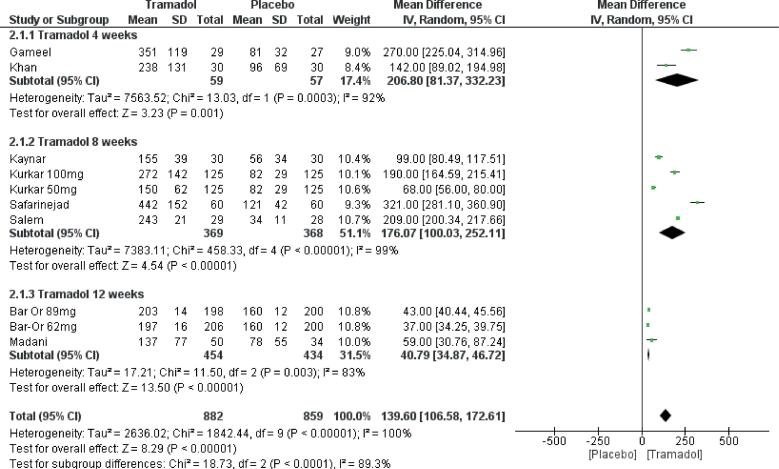
Forest plot depicting comparison of various durations of Tramadol use in premature ejaculation.

Tramadol fared better than placebo at 1 month, 2 months, and 3 months after initiation of therapy as compared to placebo (Figure-4). Gameel et al. (27) and Khan et al. (18) treatment duration was for 4 weeks and both the groups used different doses of therapy. Pooled analyses at 4 weeks revealed tramadol to be significantly better than placebo [MD 206.8 CI (81.3, 332.2), p=0.001]. Similarly at 8 weeks and 12 weeks of therapy subgroup analyses including multiple studies with different dosages of therapy revealed tramadol to be significantly better than placebo (Figure-4).

#### Tramadol vs. Paroxetine

Gameel et al. (27) and Hamidi-Madani et al. (28) compared tramadol to paroxetine. Gameel et al. (27) and Hamidi-Madani et al. (28) used 50mg on-demand tramadol and 20mg on-demand paroxetine 20mg. IELT values post-treatments were similar in the two groups with a p-value of 0.08.

#### Tramadol 100mg vs. Tramadol 50mg

**Figure-5:**
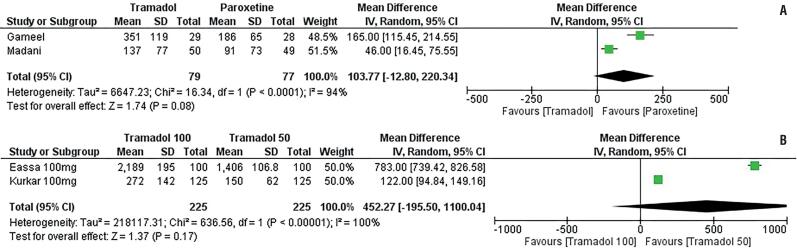
Forest plot depicting comparison of Tramadol versus paroxetine (a) and tramadol 50mg versus 100mg (b).

Only the two studies by Eassa et al. (29) and Kurkar et al. (30) compared these two doses of tramadol. The two groups of therapy were equally effective with a non-significant difference in IELT ((MD 452.7 CI [-195.5-1100], p=0.17) (Figure-5B).

#### Adverse events

**Figure-6:**
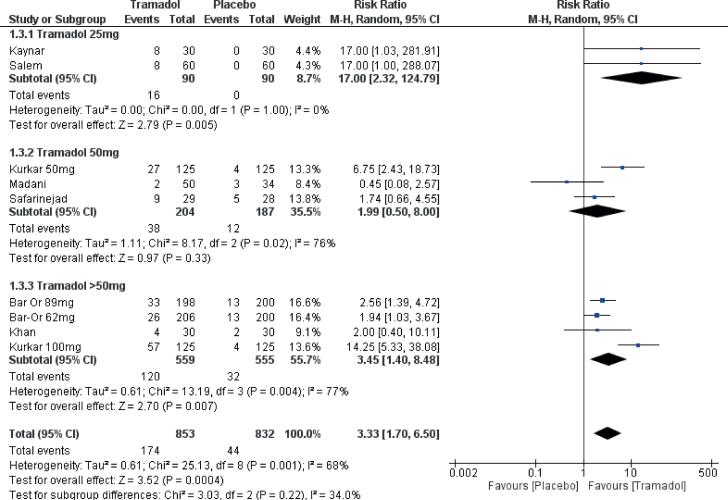
Forest plot depicting comparison of side effects of Tramadol to placebo at various doses.

Overall, tramadol had a higher incidence of adverse events as compared to placebo [risk ratio (RR) 3.3 CI (1.7, 6.5) (Figure-6). Most of these adverse events included dizziness, headache, nausea, vomiting, and constipation. None of the studies included in this review reported serious adverse events. The incidence of side effects was higher for tramadol as compared to placebo for all the doses i.e. tramadol 25mg, 50mg, >50mg. However, the difference could not reach statistical significance for the dose of 50mg.

## DISCUSSION

A variety of pharmacological agents have been tried in the management of PE ranging from topical anesthetics to SSRIs (31, 32). The use of on-demand tramadol has been shown to be efficacious in numerous studies (33, 34) albeit the apprehension regarding abuse potential and serious adverse events had limited its use in this population. Daily dosing schedule has a stronger response compared to on-demand schedule but is limited by the risk of tachyphylaxis and abuse potential giving an edge to on-demand dosing with the additional benefit of convenient dosing schedule limited to the time of maximum action needed (5). Precise mechanism of action of tramadol is not fully elucidated and has been attributed to the inhibition of serotonin and noradrenaline reuptake in the central nervous system (35). There have been very few well-conducted studies evaluating the role of tramadol in PE. Most of the initial studies have been poorly conducted open-label studies. With this study, we aimed to explore the literature pertaining to the use of tramadol in patients with PE to determine its safety and efficacy in this population. From the last review on the topic, we have added new articles published in the last 5 years in the present review (33). This meta-analysis had limitations such as the inclusion of studies, which included behavioral therapy as a control group (Xiong et al.) (10) and daily paroxetine in the comparative arm (Alghobary et al. (36)). We have also performed subgroup analysis according to the duration and dosage of Tramadol to reduce overall heterogeneity in the studies.

The overall risk of bias across the primary outcome across individual studies was unclear. Most of the studies have not addressed various domains of risk of bias tool predisposing them to bias. Adequate method of random sequence generation was provided in 3 studies only whereas most of the studies have not clearly described the technique. Apart from a study by Safarinejad et al. (37), no other study did concealment for allocation into either study group, thus were at risk for selection bias. Double-blinding i.e. blinding of patient and investigator was performed only in 4 RCT, 3 RCTs were single-blinded and the other 2 studies did not clearly define it. From risk of bias assessment, it appears that included studies were at possible risk of bias due to a number of methodological deficiencies at various levels.

Overall pooled analysis of data showed that tramadol was significantly better than the control group in improving IELT with MD of 139.6 seconds and p-value of <0.00001. The validity of this data is questionable due to associated high heterogeneity in the analysis. Heterogeneity in our opinion was due to the fact that tramadol was used in different dosages and for different duration of therapy. Despite performing subgroup analysis according to the dosage and duration of therapy heterogeneity across the studies could not be reduced.

An effective dose of tramadol balancing efficacy and side effects in not yet known. Different studies have used different doses ranging from 25mg to 100mg on-demand basis. In the present study, most of the studies used 50mg dose of tramadol, and some used 25mg and >50mg. On comparison of on-demand tramadol 25mg dose with the control group, there was no difference in IELT (MD 209.2 CI [-8.2-426.8], p=0.06). Whereas, tramadol in the 50mg and >50mg group were more effective than the placebo group. Studies by Eassa et al. and Kurkar et al., compared 100mg and 50mg doses of tramadol, pooled analysis revealed no significant difference in the two doses of tramadol in IELT (MD 452.7 CI [-195.5-1100], p=0.17). From the above data, it appears that tramadol in 50mg dose is equally efficacious to 100mg dose. However, this is only limited data obtained from two studies. There is an urgent need to conduct dose exploring studies for tramadol use in PE. However, this seems to be a distant possibility as of now due to established efficacy of other drugs such as Dapoxetine and apprehension regarding the abuse potential of tramadol (3).

Treatment duration across the studies was also a quiet variable. Treatment duration as short as 4 weeks have been reported to show significant improvements in the IELT as compared to placebo. Khan et al. in their study gave daily dosing of tramadol for 4 weeks followed by on-demand tramadol for 4 weeks without any drug-free period. They evaluated patients at 4 and 8 weeks following the start of medication. This sort of administration of tramadol makes interpretation of results very difficult. Two studies i.e. Kurkar et al and Bar-or et al. have compared various doses of tramadol for 8 and 12 weeks of duration respectively. Significant improvement in IELT has been reported compared to placebo for these varied therapies. Taken into consideration above-mentioned studies, one important question that still remains unanswered is an effective duration of therapy, both lower and upper limit of which is still not clear.

The incidence of adverse events was significantly higher in the tramadol group. Most of these adverse events included dizziness, headache, nausea, vomiting, and constipation. None of the studies included in this review reported serious adverse events. It has to be noted that studies included in this review did not explicitly study the adverse events. Abuse and dependence potential of this drug is the main deterrent of this drug becoming therapy of choice. There is evidence that dependence on this drug is common in males less than 30 years of age who consume supratherapeutic amounts of the drug and display withdrawal symptoms. This age group coincides with the population seeking treatment for PE. Many cases of dependence have been described among individuals with a history of substance abuse, long-term users, infrequent users, who consume high doses of tramadol without a history of misuse of other substances. Even though tramadol use has been described safely for other indications (38–40) with little abuse potential its use for PE will likely remain curtailed due to apprehension among the urologists regarding its long terms side effects and abuse potentials. Furthermore, the dependency potential of tramadol in “on-demand” usage for PE has not been rigorously reported in the included studies.

There are several limitations to this study. Firstly, the risk of bias in the studies included in this review appears to be a major factor limiting the strength of this meta-analysis. Serious methodological concerns in various domains have placed most of the studies at high risk of bias. Second, high heterogeneity of data across the studies due to various reasons mentioned previously makes the interpretation of data difficult. Single blindness, lack of proper allocation concealment, cross over study designs, administration of daily dose followed by on-demand dosage, and existence of behavioral therapy group as control not only adds to heterogeneity across the studies also jeopardizes the results of the study.

## CONCLUSIONS

Tramadol appears to be an effective drug for the management of PE with a low propensity for serious adverse events. However, effective duration and dose of therapy are not known. Further good-quality studies are needed with adequate data on dosage, duration of therapy, and long term data on the side effect profile of tramadol. Further studies are warranted comparing tramadol to other drugs before recommending the widespread use of tramadol.

## ABBREVIATIONS

PE: Premature ejaculation
PRISMA: Preferred reporting Items for Systematic reviews and Meta-analysis
RCT: Randomized controlled trials
ISSM: International society of sexual medicine
IELT: Intravaginal ejaculation latency time
DSM: Diagnostic and Statistical Manual of Mental Disorders
CI: Confidence interval
MD: Mean difference
SSRI's0: Selective serotonin uptake inhibitors
RR: Risk ratio
